# Molecular dynamics simulations suggest why the A2058G mutation in 23S RNA results in bacterial resistance against clindamycin

**DOI:** 10.1007/s00894-018-3689-5

**Published:** 2018-07-03

**Authors:** Katarzyna Kulczycka-Mierzejewska, Joanna Sadlej, Joanna Trylska

**Affiliations:** 1 Interdisciplinary Centre for Mathematical and Computational Modelling, University of Warsaw, Pawinskiego 5A, 02-106 Warsaw, Poland; 2 Faculty of Chemistry, University of Warsaw, Pasteura 1, 02-093 Warsaw, Poland; 3 Centre of New Technologies, University of Warsaw, Banacha 2c, 02-093 Warsaw, Poland

**Keywords:** Clindamycin, 23S ribosomal RNA, Molecular dynamics simulations, A2058G mutation, Bacterial resistance

## Abstract

Clindamycin, a lincosamide antibiotic, binds to 23S ribosomal RNA and inhibits protein synthesis. The A2058G mutation in 23S RNA results in bacterial resistance to clindamycin. To understand the influence of this mutation on short-range interactions of clindamycin with 23S RNA, we carried out full-atom molecular dynamics simulations of a ribosome fragment containing clindamycin binding site. We compared the dynamical behavior of this fragment simulated with and without the A2058G mutation. Molecular dynamics simulations suggest that clindamycin in the native ribosomal binding site is more internally flexible than in the A2058G mutant. Only in the native ribosome fragment did we observe intramolecular conformational change of clindamycin around its C7-N1-C10-C11 dihedral. In the mutant, G2058 makes more stable hydrogen bonds with clindamycin hindering its conformational freedom in the ribosome-bound state. Clindamycin binding site is located in the entrance to the tunnel through which the newly synthesized polypeptide leaves the ribosome. We observed that in the native ribosome fragment, clindamycin blocks the passage in the tunnel entrance, whereas in the mutated fragment the aperture is undisturbed due to a different mode of binding of clindamycin in the mutant. Restricted conformational freedom of clindamycin in a position not blocking the tunnel entrance in the A2058G mutant could explain the molecular mechanism of bacterial resistance against clindamycin occurring in this mutant.

**Electronic supplementary material:**

The online version of this article (10.1007/s00894-018-3689-5) contains supplementary material, which is available to authorized users.

## Introduction

The ribosome is a macromolecular complex built from ribosomal ribonucleic acid (rRNA) and proteins. It is composed of two subunits, small and large (in bacteria termed 30S and 50S, respectively) [1]. The bacterial ribosome, as being essential for protein synthesis in bacterial cells, is targeted by many antibiotics.

**Fig. 1:**
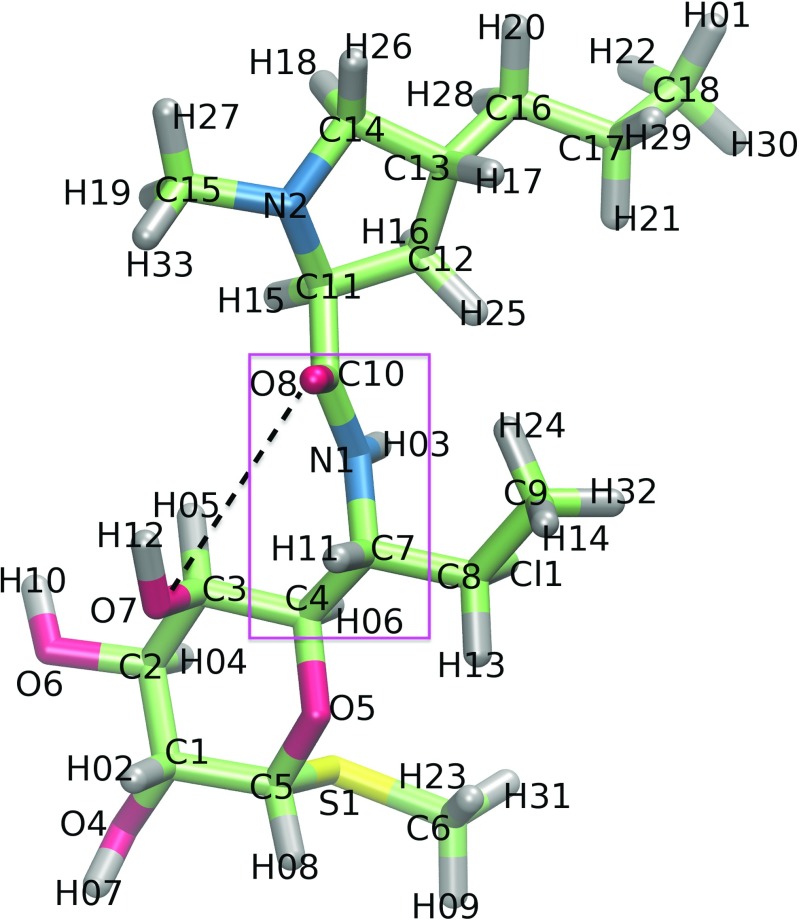
The model of clindamycin with atom numbering as used in the text; C: *cyan*, O: *red*, N: *blue*, S: *yellow*, H: *grey*. The intramolecular hydrogen bond between O7 and O8 atoms is schematically marked with a *black dashed line* and the dihedral angle enabling the formation of two clindamycin conformers is in a *pink frame*

Several classes of antibacterial agents (for example aminoglycosides, lincosamides, macrolides, streptogramins B) bind to the ribosome and affect protein synthesis [2]. Lincosamides are a group of antibiotics consisting of three compounds. One of the compounds is a natural lincomycin. The other two, clindamycin and pirlimycin, are semi-synthetic derivatives of lincomycin. Clindamycin is shown in Fig. 1. These compounds inhibit bacterial protein synthesis by blocking the catalytic ribosomal peptidyl transferase center (PTC), which is responsible for the formation of peptide bonds joining the amino acids. Binding of lincosamides to PTC prevents proper orientation of tRNA and interferes with peptide bond formation in the newly created polypeptide chain [3]. PTC is also connected with a tunnel in the 50S subunit through which the nascent polypeptide travels to finally leave the ribosome complex.

Lincosamides are known for their effectiveness against many anaerobic bacteria (e.g., *Bacteroides fragilis*) and Gram-positive bacteria (e.g., *Streptococcus*). They also serve as antimalarial drugs because they are active against the *Plasmodium* parasite. Gram-negative bacteria are resistant to lincosamides apart from *Capnocytophaga canimorsus* [4]. Clindamycin can be also effective against methicillin-resistant *Staphylococcus aureus* (MRSA) in skin and soft tissue infections [5].

Unfortunately, decades of clinical use of lincosamides have led to an increase of drug resistance in many bacterial strains. There are several known resistance mechanisms acquired by bacteria against lincosamides, including active efflux from the cell and enzymatic deactivation of the compound [6]. The resistance can also arise from various modifications of lincosamides’ ribosome target such as methylation of 23S RNA base no. 2058 [7–9] and mutations of G2057, A2058, A2059, C2452, and C2611 [10]. Just one methylation at a certain position of 23S rRNA leads to resistance to lincomycin and clindamycin [8]. Due to methylation, the minimal inhibitory concentrations required to inhibit bacterial growth increase from 2 to 512 *μ* g/ml for clindamycin and from 16 to 8000 *μ* g/ml for lincomycin. Bacteria resistant to lincosamides also exhibit resistance to macrolides [11–13] with a similar mechanism, i.e., due to modifications of A2058. In addition to bacterial resistance, lincosamides also have side effects such as diarrhea, nausea, rash, hypersensitivity, and jaundice [14, 15], which further limit their efficiency in therapy.

Therefore, understanding the mechanisms that govern bacterial resistance on a molecular level is needed to be able to improve lincosamide selectivity and reduce their side effects. Fortunately, structural data of some lincosamides and their targets are known. The structures of free lincosamides have been examined using experimental methods: X-ray techniques [16, 17] and ^13^C NMR spectroscopy [18], as well as computational techniques: quantum calculations [19] and molecular dynamics (MD) [18, 20].

Lincosamides are internally flexible and acquire different conformations upon binding to their molecular target. Two-dimensional transferred nuclear Overhauser effect spectroscopy (TRNOESY) investigations together with molecular dynamics simulations showed that lincosamide’s conformation is important for binding to the ribosome target [18]. Also, there are three crystal structures of clindamycin resolved in the complex with the ribosome [21–23], and one crystal structure in the complex with the LinB protein [24]. The LinB protein is a bacterial inactivating enzyme catalyzing adenylylation of lincosamides. The conformations of clindamycin in these complexes differ among the structures by a 180° rotation of the pyrrolidynyl propyl group (Fig. 1). This happens because clindamycin can form an intramolecular hydrogen bond [19]. Indeed, Kostopoulou et al. [25] using foot printing experiments and computational docking showed two clindamycin binding positions within the PTC.

In our previous work [19], at the B3LYP level of theory with the 6- 31G** basis set, we characterized clindamycin, lincomycin, and pirlimycin conformers both in vacuum and surrounded by point charges mimicking the electrostatic field of the ribosome. Combining the results of the natural bond orbital analysis and atoms in molecules theory, we described intramolecular hydrogen bonds in the above lincosamides. In [20], we focused on clindamycin and with Born-Oppenheimer, quantum-mechanical, and classical full-atom molecular dynamics simulations, we analyzed the transitions between its two conformers.

The goal of this work was to examine internal flexibility of clindamycin binding site in the wild-type and A2058G mutated bacterial ribosome fragment. We aimed to understand why this single-point mutation results in blocking the antibacterial activity of clindamycin. Since the influence of the same ribosome modification but on a different antibiotic, telithromycin from the ketolide class, has been previously successfully investigated with molecular dynamics methods [26] we also applied this computational technique.

## Methods

### Structure preparation

**Fig. 2:**
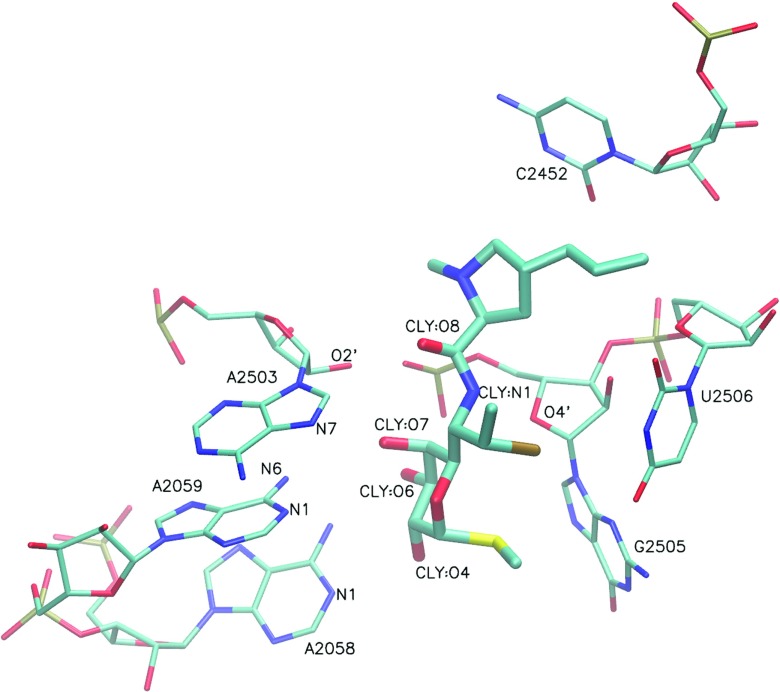
Nucleotides in proximity to clindamycin (CLY). For clarity, only heavy atoms are shown in an orthographic representation. The conformation is from the 4V7V structure [23]

Three structures of clindamycin complexed with bacterial ribosome were available in the Protein Data Bank (PDB [27]) as of Nov 2015. Their PDB codes are 1JZX [21], 1YJN [22], and 4V7V [23]. The 1JZX structure contains a higher-energy conformer of clindamycin, while the 1YJN and 4V7V contain similar low-energy conformers (Fig. 2). The root mean square deviation (RMSD) for clindamycin (calculated for heavy atoms) between the 1YJN and 4V7V structures equals 0.22 Å. However, the 1YJN structure comes from an extremophile bacteria *Haloarcula marismortui* and involves a G2099A mutant. The 4V7V structure is from *Escherichia coli* bacterium, which made it most suitable for our purpose and was selected for this study.

For simulations, all residues within a sphere with the radius of about 20 Å around clindamycin were cut out of the ribosome structure (Fig. S1). All rRNA and protein chains shorter than three residues were then removed. The obtained sphere contained 23S rRNA (353 nucleotides), L3, L16, L22, L32 ribosomal protein (82 amino acids), one clindamycin molecule, 39 magnesium ions, and 170 crystal water molecules. Hydrogen atoms were added with tleap [28]. A total of 228 K^+^ ions were added to neutralize the charge. The system was solvated with approximately 25,000 TIP3P [29] water molecules arranged in a truncated octahedron shape around the solute, adding 15 Å to its size. The end result was an almost 90,000-atom system. Besides the fragment of the ribosome complexed with clindamycin (termed WT–CLY), a free fragment (WT) of the ribosome was also simulated. Our investigation also included a complex of clindamycin with a mutated ribosome (MUT–CLY) and a mutated free ribosome fragment (MUT). The four simulated systems are described in Table 1. All structure preparations were done using PyMol [30].

**Table 1:** MD simulation types and their abbreviations used in the text

Simulated system	Abbreviation	Average RMSD [Å]	Average R_*G*_ [Å]
Wild-type ribosome fragment with clindamycin	WT–CLY-1	2.18 ± 0.03	28.88 ± 0.04
	WT–CLY-2	2.16 ± 0.03	28.86 ± 0.04
	WT–CLY-3	2.19 ± 0.03	28.91 ± 0.03
	WT–CLY-4	2.26 ± 0.04	28.88 ± 0.04
A2058G mutated ribosome fragment with clindamycin	MUT–CLY-1	3.01 ± 0.04	29.40 ± 0.04
	MUT–CLY-2	3.05 ± 0.04	29.40 ± 0.03
	MUT–CLY-3	3.02 ± 0.03	29.40 ± 0.03
Wild-type ribosome fragment	WT-1	2.51 ± 0.04	29.12 ± 0.04
	WT-2	2.49 ± 0.07	29.15 ± 0.03
	WT-3	2.49 ± 0.05	29.26 ± 0.04
A2058G mutated ribosome fragment	MUT-1	2.64 ± 0.05	29.05 ± 0.03
	MUT-2	2.59 ± 0.06	29.00 ± 0.03
	MUT-3	2.63 ± 0.05	29.00 ± 0.03

The average RMSD values (from the production trajectory but relative to the starting structure) and average R_*G*_ values (both with standard deviation) are calculated for the C_*α*_ and P atoms. The simulated system is shown in Fig. S1

### Molecular simulation set-up and protocol

Amber ff10 force field containing the bsc0 [31] and glycosidic bond torsion corrections [32] were used. The standard Amber Lennard–Jones parameters were applied for the K^+^ ion with the radius of 1.705 Å and well depth of 0.1936829 kcal/mol [33]. The parameters for Mg^2+^ were taken from [34]. Clindamycin force field was obtained using Antechamber [28]. The partial RESP charges at the B3LYP/6-31G** level [35, 36] were calculated with GAUSSIAN 09 [37]. Testing of the parameters was performed by running a 300-ns MD simulation of clindamycin in explicit water [20].

Three-stage minimization with AMBER12 [28] was used for relaxation of all systems. First, only the solvent hydrogen atoms were minimized. Second, minimization was applied to all water molecules. Third, all solute heavy atoms, all solvent molecules, and ions were minimized. In each stage, minimization was performed with 2000 steps of steepest descent method followed by 3000 steps of conjugate gradient method. A 50 kcal/(mol⋅Å^2^) force constant was applied to restrain atoms not undergoing minimization.

Simulations were performed with the NAMD package [38]. Periodic boundary conditions were used with electrostatic interactions calculated with the Particle Mesh Ewald method [39]. Temperature was regulated by the Langevin thermostat [39]. The SHAKE algorithm [40] was applied to facilitate time step extension to 2 fs.

Heating of the system was done in 200-ps-long thermalization, according to ref. [41]. The temperature was increased from 0 to 310 K. During heating, constraints were applied to rRNA and protein atoms, Mg^2+^ ions, and crystal water molecules—these parts were treated as solute. For the first 150 ps of thermalization, the force constant was set to 50 kcal/(mol⋅Å^2^) and the temperature was increased by 30 K for 10 ps and then by 20 K every 10 ps until it achieved 310 K. For the next 10 ps, the simulation was performed with constant temperature. During the following 40 ps, the simulation continued with the force constant decreased by 50%.

The equilibration stage comprised two parts. The first involved reducing the constraints by 50% in six runs, lasting 50 ps each, until the constraints reached 0.15625 kcal/(mol⋅Å^2^). To avoid the end effects and mimic the ribosome environment more precisely, during the second part of the equilibration and production stages, the atoms that were originally in contact with other 50S subunit residues (as in the crystal structure) were constrained with the following formula:

$$k = \left\{\begin{array}{llllllllllllll} n * \frac{0.35}{20}, & \text{for } n \leq 20 \\ 0.35, & \text{for } n > 20 \end{array}\right. $$

where *k* is the force constant and *n* is the number of the original contacts in the 50S subunit. Other atoms were not restrained. The structure of the simulated ribosome fragment colored by the *k* values applied during the production stage is shown in Fig. S2. Finally, four 100-ns production stage runs were performed for the WT–CLY system and three for the MUT–CLY, WT, and MUT systems each. The simulation time totaled 1.3 *μ* s.

### Data analysis

Unless explicitly stated, a 100-ns-long production phase was used in all analyses. The following properties were analyzed with Amber Tools suite [28]: root mean square deviation (RMSD), radius of gyration (R_*G*_), and root mean square fluctuation (RMSF). The criteria for initial screening and detection of possible hydrogen bonds were as follows: the maximum distance between the donor and acceptor was no more than 4 Å and the acceptor–proton–donor angle was between 145° and 180°. Hydrogen bonds were analyzed with MINT [42]. RNA hydrogen bond patterns based on the nucleotide edge-to-edge classification [43, 44], base pair geometric isomerism, motifs, and aromatic stacking over simulation time were analyzed with MINT [42]. Glycosidic bonds in nucleotide pairs were checked for *cis*–*trans* isomerism. The stacking energy of two nucleobases was estimated as their van der Waals (vdW) interaction and two bases were assumed stacked if the vdW energy was lower than -0.5 kcal/mol [42]. VMD [45] was used to visualize the trajectories. Graphs were prepared with Grace [46].

## Results

### Global stability measures

We carried out thirteen 100-ns-long, full-atom (MD) simulations of the bacterial ribosome fragment cut out from the 50S subunit. The simulations were performed both for the native fragment and with the A2058G mutation and also either with or without clindamycin. The free systems are termed WT (wild-type ribosome fragment), the systems with the A2058G mutation—MUT, and systems complexed with clindamycin—WT-CLY and MUT-CLY (Table 1).

**Fig. 3:**
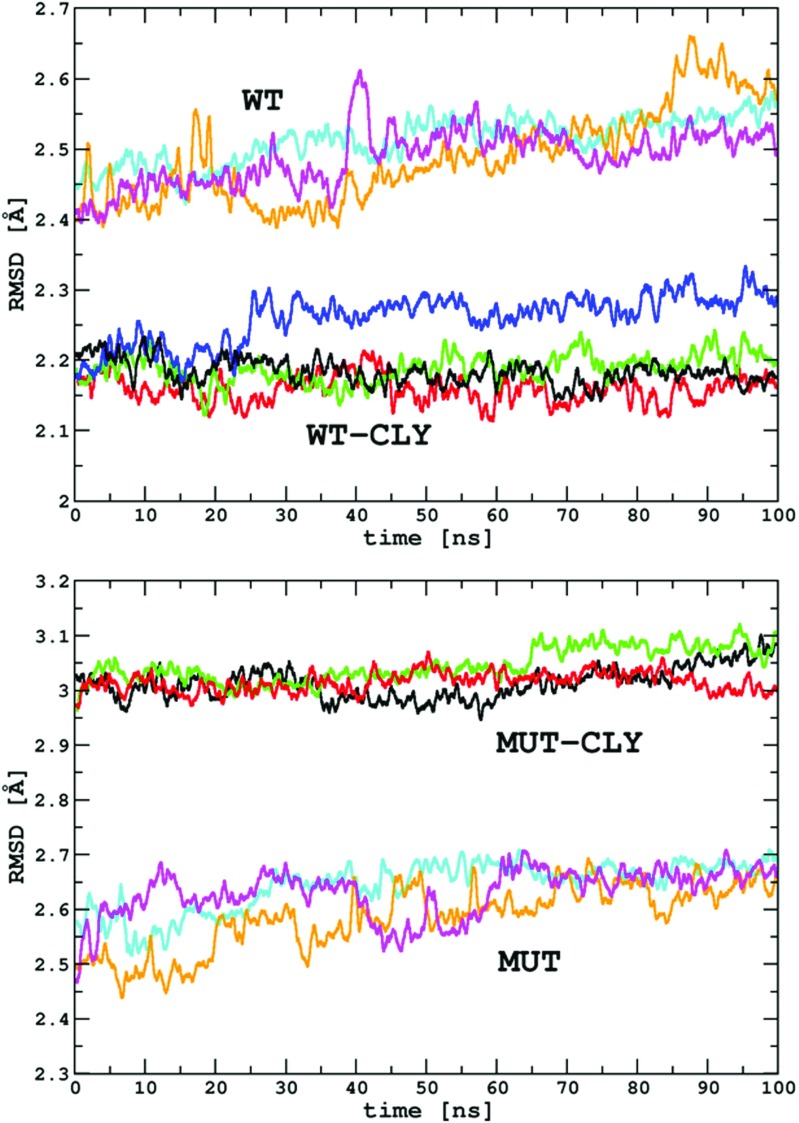
The RMSD [Å] for the C_*α*_ and P atoms calculated with respect to the starting structure. RMSD is plotted as a function of simulation time in various production runs. Data are shown as running averages over 100 points. *Lines* corresponding to the same systems are labeled WT, WT-CLY, MUT, and MUT-CLY and explained in Table 1

The RMSD for the solute *C*_*α*_ and P atoms calculated relative to starting structures is presented in Table 1 and Fig. 3. The RMSD in the WT and MUT production simulations fluctuated around 2.5 Å and 2.6 Å, respectively. The MUT–CLY simulations show about 0.8 Å higher average RMSD than the WT–CLY simulations, suggesting some influence of clindamycin on the mutated system.

The R_*G*_ in free systems was stable throughout all simulations (Table 1 and Fig. S3). Small fluctuations observed along the trajectories (with standard deviation up to 0.04 Å) were due to rearrangements of the residues on the solvent-exposed surface. The fluctuations of R_*G*_ in the systems complexed with clindamycin are again only up to 0.04 Å (Fig. S3).

### A2058G mutation affects average fluctuations of clindamycin binding site

**Fig. 4:**
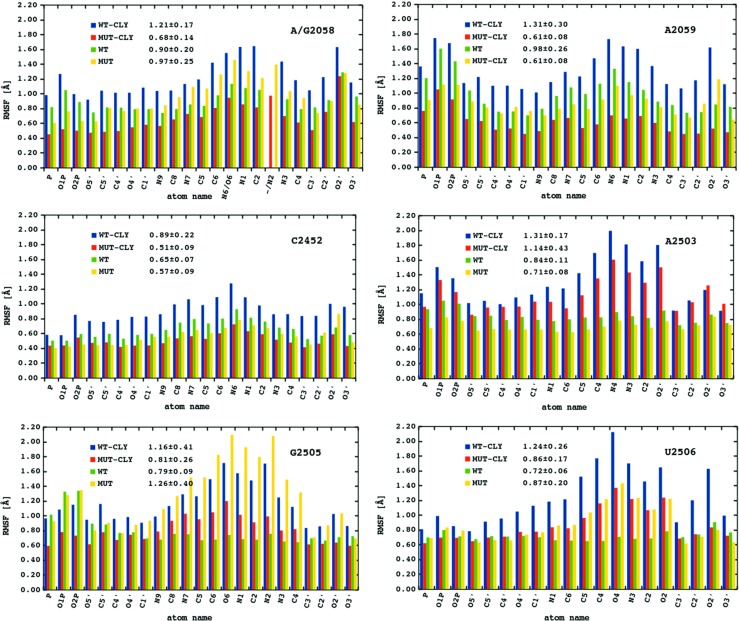
The RMSF [Å] calculated for the heavy atoms (and the average RMSF) of selected nucleotides neighboring the mutation and clindamycin binding site for each type of simulation

The A2058G mutation did not affect the average RMSF of the whole ribosome fragment, but the mutation affected the fluctuations of bases in or near clindamycin binding site (Fig. S4). Therefore, for selected nucleobases adjacent to the mutation site, we analyzed the fluctuations in more detail (Fig. 4 and Table S1).

The differences in average RMSF between the WT and MUT simulations are below 0.4 Å and only for the G2505 nucleotide are about 0.5 Å (compare green and yellow bars in Fig. 4). For the systems in the complex with clindamycin the differences between WT–CLY and MUT–CLY are more pronounced (compare red and blue bars in Fig. 4). The largest differences between WT–CLY and MUT–CLY were between A2058 (the A→G mutated nucleotide) and A2059 (its nearest neighbor in the chain), on average about 0.5 and 0.7 Å, respectively. Overall, the mutation affects the fluctuations of the clindamycin binding site to a larger extent in the systems complexed with the antibiotic.

### Average fluctuations of clindamycin are lower in the MUT–CLY system

**Fig. 5:**
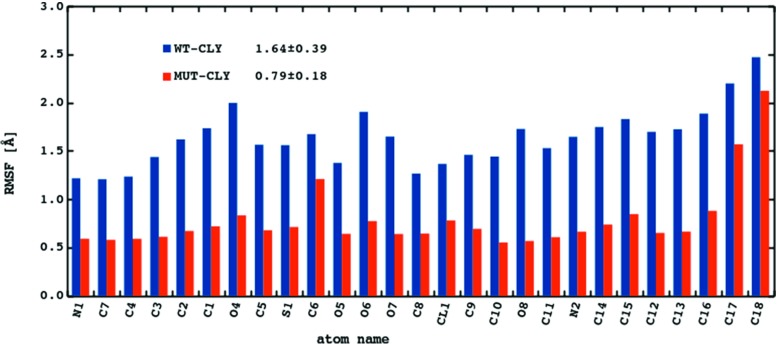
The average RMSF [Å] calculated for the heavy atoms of clindamycin. For atom names see Fig. 1

Figure 5 shows the RMSF calculated for clindamycin heavy atoms. The fluctuations of clindamycin atoms in the WT–CLY system are on average about 0.8 Å higher than in the MUT–CLY system, suggesting that clindamycin is more conformationally stable in the binding site of the mutant. This difference is probably related to the size of the clindamycin binding cleft because the cavity in the mutated system is spatially smaller and does not allow for clindamycin conformational change, which is discussed further. Overall, in all systems, the most flexible antibiotic part is the propyl group (C16-C17-C18, for atom names see Fig. 1). The methyl (C15), hydroxyl (O4 and O6), and chloroethyl chain (Cl, C8, and C9) also fluctuate noticeably.

### Conformational change of clindamycin in the WT–CLY complex

**Fig. 6:**
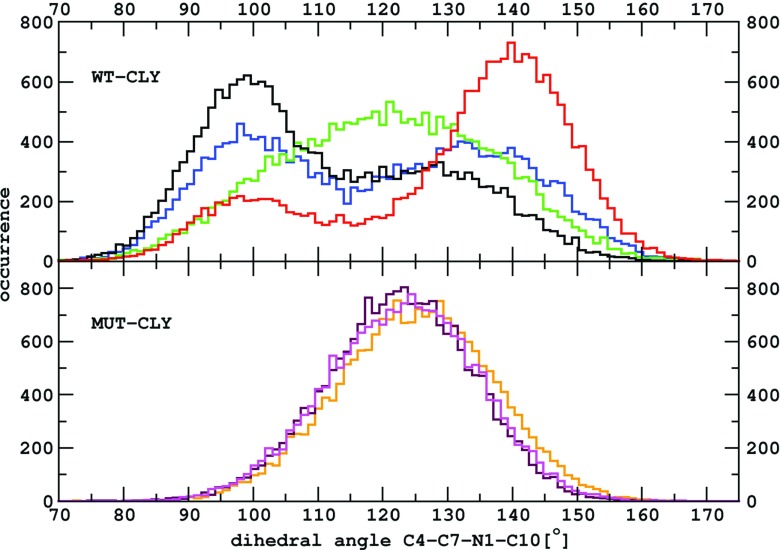
The histograms of the C4-C7-N1-C10 angle values derived from WT–CLY and MUT–CLY simulations. *Colors* correspond to simulation types listed in Table 1

Average fluctuations of the O8 atom of the carbonyl group are higher by over 1 Å in the WT–CLY than in MUT–CLY simulations (Fig. 5). This is a result of a reversible change of the C4-C7-N1-C10 dihedral angle in the WT–CLY system (Fig. 1). The conformational change of this dihedral from about 100° to 140° is shown in the histograms of Fig. 6. This intramolecular change did not occur in any of the MUT–CLY simulations, only in WT–CLY. The occurrence of this angle is single peaked in MUT–CLY simulations with a maximum between 120° and 130°.

As suggested by the crystal structures [21–23] and foot printing and docking studies [25], clindamycin may acquire two forms in the ribosome binding site and it seems the transition between these conformers has been observed by us with classical MD. Such change in the C4-C7-N1-C10 dihedral angle was also found in our previous work [20], in which we simulated clindamycin in vacuum using Born–Oppenheimer MD and in a box of water molecules using all-atom MD. However, the ranges of angles were different than obtained here in Fig. 6: on average from -41° to ca. 95°, in the case of Born–Oppenheimer MD and from -27° to ca. 123°, in the case of MD of free clindamycin in explicit solvent. These differences could be due to different conformers used as starting structures in our previous work [20] or the fact that in this work we included the ribosome context that affects clindamycin’s conformational freedom.

### Clindamycin binding cavity

**Fig. 7:**
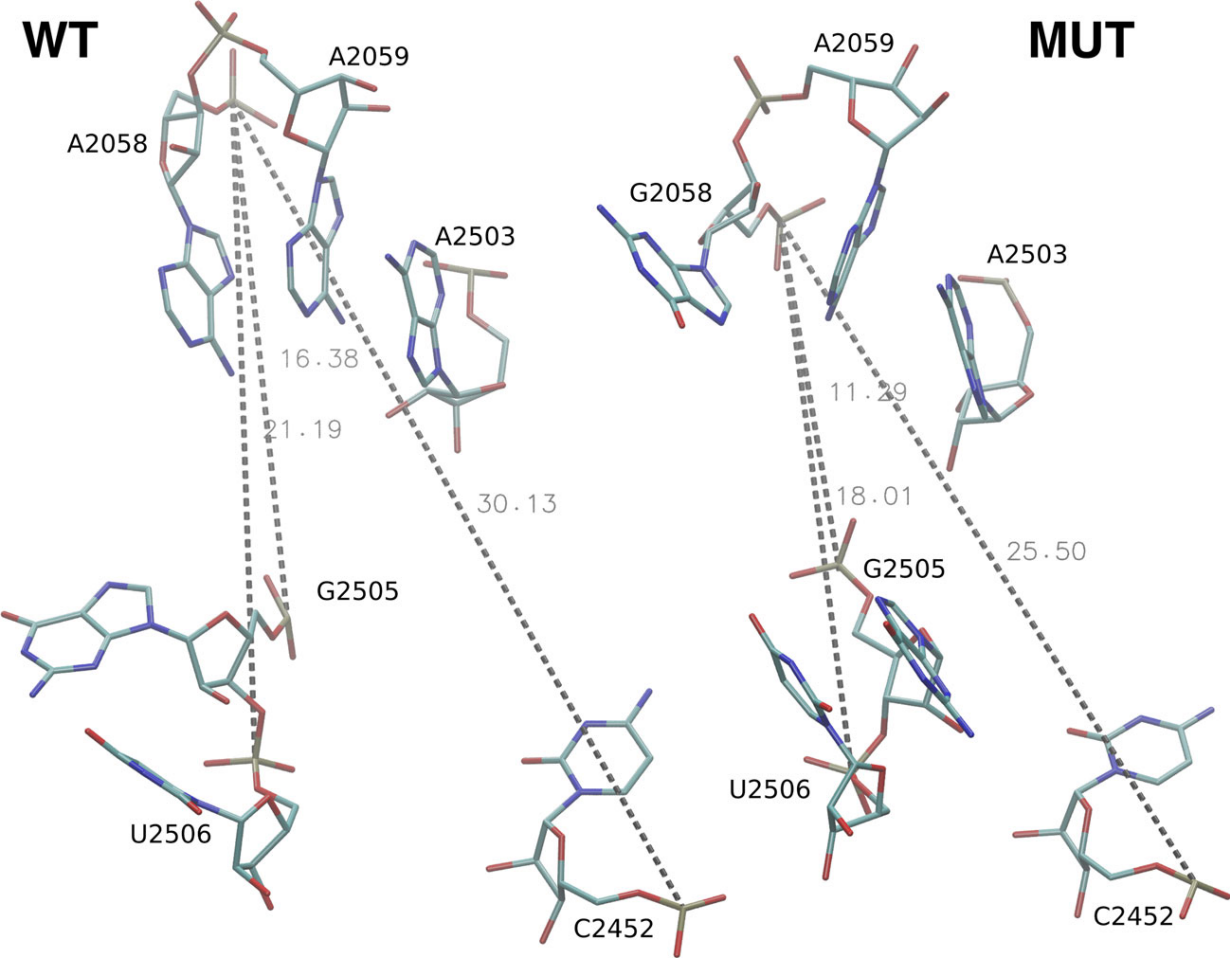
Nucleotides of clindamycin binding pocket with marked P–P distances as in the snapshots found in the WT and MUT trajectories. The distances are in Å

Table 2 shows the average P–P distances between nucleobases comprising the clindamycin binding site and Fig. 7 the P–P distances in trajectory snapshots. In some cases in the MUT system, the P–P distances are on average from 1 to 3 Å smaller than in the WT system (compare WT and MUT columns in Table 2). For example, the distance between the P atoms of A/G2058 and A2452 in the MUT system is about 3 Å smaller than in the WT system. This suggests that the mutation changes the phosphate group positions in the clindamycin binding site making the MUT cavity on average slightly smaller than in the WT system.

**Table 2:** Distances (with standard deviations) between P atoms of selected nucleotides averaged over all trajectories of a given type

Nucleotide	P–P distance [Å]			
	WT–CLY	MUT–CLY	WT	MUT
A/G2058-C2452	28.3 ± 1.2	31.3 ± 0.2	29.8 ± 0.6	26.7 ± 0.4
A/G2058-G2505	12.4 ± 0.1	14.9 ± 0.3	14.7 ± 0.6	12.4 ± 0.5
A/G2058-U2506	18.1 ± 0.7	19.8 ± 0.1	20.6 ± 0.4	18.5 ± 0.7
A2059-C2452	29.1 ± 1.6	32.2 ± 0.3	30.4 ± 1.0	28.5 ± 0.6
A2059-G2505	15.1 ± 0.4	17.6 ± 0.4	15.6 ± 1.1	15.1 ± 1.0
A2058-U2506	19.2 ± 1.3	21.9 ± 0.2	21.4 ± 0.7	20.7 ± 1.3
A2503-C2452	20.9 ± 0.8	24.0 ± 0.3	22.1 ± 0.3	21.6 ± 0.3
A2503-G2505	10.6 ± 0.3	11.9 ± 0.2	10.8 ± 0.1	10.8 ± 0.4
A2503-U2506	15.7 ± 0.2	16.9 ± 0.1	16.9 ± 0.6	16.9 ± 0.2

On the contrary, once clindamycin is bound, the P–P distances in MUT–CLY are on average higher (by 1.5 to 3.5 Å) than in WT–CLY. This observation, in principle, does not agree with smaller fluctuations of clindamycin in the MUT–CLY ribosome fragment (Fig. 5) and suggests a different mode of binding of clindamycin in the mutated system, which is discussed below.

### Clindamycin affects the ribosome tunnel entrance

**Fig. 8:**
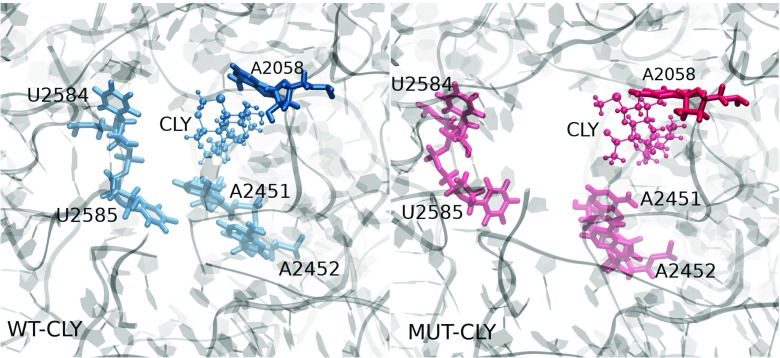
Snapshots from the WT–CLY (*left*) and MUT–CLY (*right*) trajectories showing the position of clindamycin and the nearby bases with respect to ribosome tunnel entrance rendered from the same viewpoint. The ribosome fragment is depicted as transparent *grey ribbons*

**Fig. 9:**
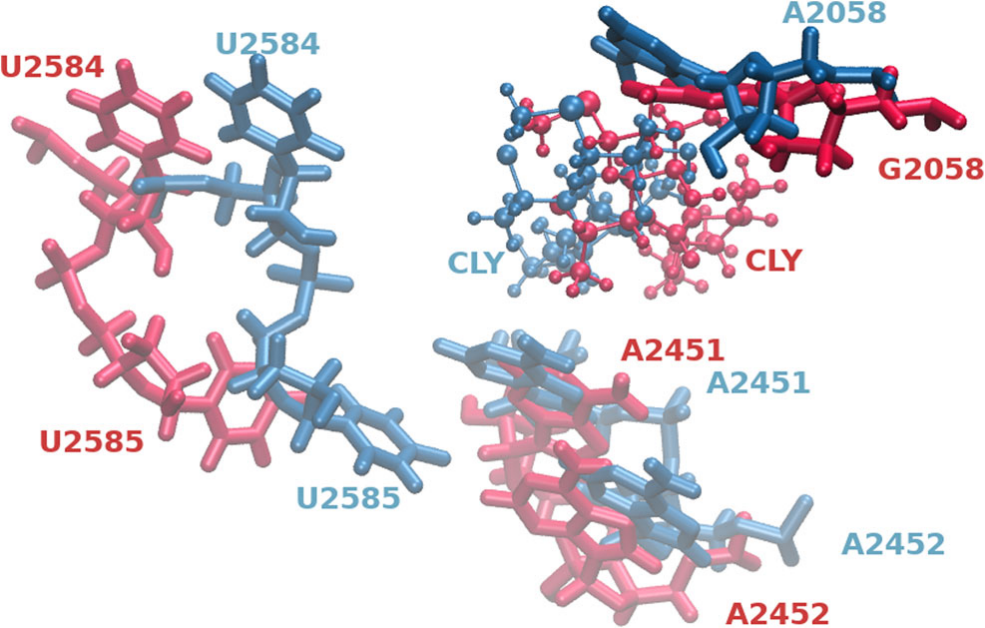
Snapshots of clindamycin and the neighboring bases from the WT-CLY (*blue*) and MUT-CLY (*red*) trajectories superposed according to the A2059 base

The ribosome exit tunnel is the pathway in the large subunit through which the newly synthesized polypeptide leaves the ribosome. The binding site area of clindamycin shown from the direction of the entrance to the exit tunnel is presented in Fig. 8. In the WT–CLY system, the entrance to the exit tunnel is narrow contrary to the wider space visible in the MUT–CLY system (compare left and right image in Fig. 8). A superposition of the binding site area from both trajectories viewed from the direction of the exit tunnel is shown in Fig. 9 to better illustrate the differences in spatial position of clindamycin between the native and mutated ribosome. Clearly, the positioning of clindamycin is different in the mutant and allows for opening of the entrance to the exit tunnel. This fact may help understand, at a molecular level, why clindamycin, even though bound to the ribosome mutant, does not change the activity of the ribosome (polypeptides are still free to leave the ribosome). At the macromolecular scale, a result of such behavior appears as a resistant bacterial strain.

**Fig. 10:**
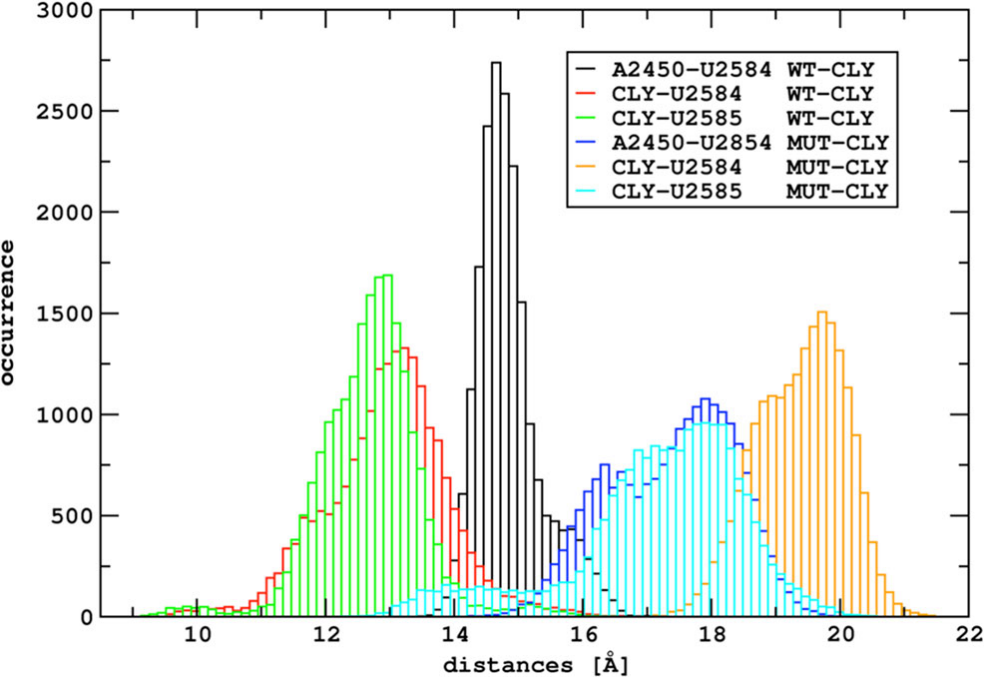
The histograms of the A2450–U2584, CLY–U2584 and CLY–U2585 center of mass distances obtained from WT–CLY and MUT–CLY simulations

Thus, the spatial orientation of the binding site, as well as its neighborhood, changes as an effect of the A2058G mutation. The nucleobases that are involved in the binding of peptidyl-tRNA to the ribosome are A2451, A2506, U2584, and U2585, and of aminoacyl-tRNA – A2451 [47]. The distances between the centers of masses of these bases and clindamycin are larger by a few Å in the MUT–CLY system in comparison with the WT–CLY system (see Fig. 10). The differences in these distances between selected nucleobases and clindamycin, shown in Table S2, again confirm the image of Fig. 8. In summary, in the MUT–CLY system, the ribosome tunnel entrance is larger than in the WT–CLY system.

### Clindamycin intramolecular hydrogen bond

Crystallographic data [21, 22] suggest six bases: A/G2058, A2059, C2452, A2503, G2505, and U2506, which are important for clindamycin interactions with the ribosome (Fig. 2). The most frequent hydrogen bonds formed between these bases and clindamycin, as well as intramolecular hydrogen bond within clindamycin, derived from our trajectories are shown in Table 3.

**Table 3:** Intramolecular hydrogen bond between O7 and O8 in clindamycin (observed only in WT–CLY trajectories) and intermolecular hydrogen bonds between clindamycin and nucleotides occurring over 10% of total simulation time

Sim. type		Donor	Acceptor	% of sim.	D ⋯ A	D-H ⋯ A
and number		(D)	(A)	time	[Å]	[°]
WT–CLY	1	CLY:O7	CLY:O8	54	2.74 ± 0.06	158.9 ± 3.1
	2			13		
	3			36		
	1	A2059:N6	CLY:O4	29	3.05 ± 0.04	155.1 ± 1.1
	2			34		
	3			58		
	1	CLY:O4	A2059:N1	23	2.91 ± 0.01	158.6 ± 1.9
	2			35		
	3			27		
MUT–CLY	1	G2058:N1	CLY:O4	27	3.02 ± 0.02	146.0 ± 2.8
	2			29		
	3			25		
	1	CLY:O4	G2058:O6	82	2.71 ± 0.03	158.2 ± 2.2
	2			87		
	3			78		
	1	A2059:N6	CLY:O6	74	3.11 ± 0.04	152.9 ± 1.2
	2			86		
	3			84		
	1	CLY:O6	A2059:N1	38	2.90 ± 0.02	157.8 ± 0.9
	2			64		
	3			40		
	1	CLY:O7	A2503:O2’	86	2.79 ± 0.01	161.3 ± 1.3
	2			96		
	3			92		
	1	CLY:N1	G2505:O’4	61	3.51 ± 0.03	158.4 ± 0.9
	2			92		
	3			82		

The simulation types are listed in Table 1. For atom names see Figs. 1 and 2

In three out of four WT–CLY trajectories, we observed an internal hydrogen bond in clindamycin marked in Fig. 1. This O7–H12⋯O8 bond was present on average 34% of time, with the average O7–O8 distance of 2.74 ± 0.06 Å and O7–H12⋯O8 angle of 158.9 ± 3.1. As previously calculated, this hydrogen bond assures a lower energy conformer of clindamycin in vacuum and water environment [19]. However, in WT–CLY simulations, we also observed a change to a higher-energy conformer with respect to the dihedral angle C4-C7-N1-C10 (see previous sections), suggesting that there might be a connection between the internal O7–H12⋯O8 hydrogen bond in clindamycin and conformational change of its C4-C7-N1-C10 dihedral angle (Fig. 1). This observation is in accord with two conformers of clindamycin observed in crystal structures [21–23] and docking studies [25], suggesting that there is room for clindamycin to rotate its pyrrolidynyl propyl group even once bound in the ribosome.

### Clindamycin hydrogen bonds with the 2058 and 2059 bases

**Fig. 11:**
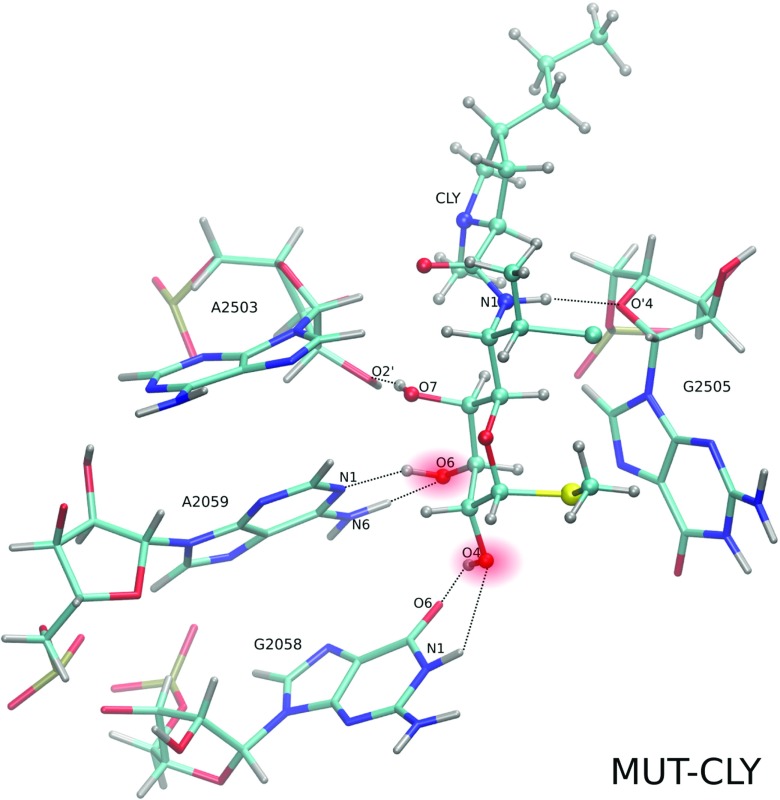
Trajectory snapshot presenting the hydrogen bonds (*dashed lines*) between CLY and neighboring nucleobases in the MUT–CLY system

Clindamycin hydrogen bond network with the 2058 base depends on the base type. In the mutant, if G2058 is present, two types of hydrogen bonds between CLY:O4 and G2058 are formed (Fig. 11). One of them, G2058:O6⋯CLY:O4, was particularly stable, on average, for 82 ± 5% of simulation time (Table 3). The second one, G2058:N1⋯CLY:O4, was observed for about 25% of time.

If wild-type A2058 is present, the occurrence of clindamycin intramolecular interactions makes its contacts with nucleobases more transient. One hydrogen bond between CLY and A2058 is preferentially formed, A2058:N1⋯CLY:O4, but it was observed for about 7% of simulation time. This is because in the WT–CLY system the CLY:O4 atom engages in the interactions also with A2059; with A2059:N6 atom for about 40 ± 15% of time and A2059:N1⋯CLY:O4 for about 29 ± 6% of time.

Replacing A with G at 2058 position also changes the interactions with A2059 (Fig. 11). In the mutated complexed systems, the hydrogen bonds with A2059 are formed not via O4 but via O6 atom of clindamycin (CLY:O6⋯A2059:N6 – 81 ± 6% simulation time and A2059:N1⋯CLY:O6 – 47 ± 14%). Therefore, in the mutant, the position of clindamycin is shifted in comparison with the WT–CLY system. Overall, in the WT-CLY system the hydrogen bonds that CLY forms are more transient with higher residual fluctuations and CLY may change its conformation, which does not hold for the MUT–CLY system.

### Hydrogen network pattern among nucleobases

We further analyzed the hydrogen bond network among nucleotides forming the clindamycin binding site. The selected nucleotide pairs and the percent of simulation time that they are formed is shown in Table S3. The highest number of pairs was observed for the WT system. Also, the two pairs in the WT system, C2452–U2504 and U2506–G2586, were present for over 75*%* of simulation time. Binding of the antibiotic to the native structure, as expected, reduces the amount of nucleotides interacting with each other because some of them interact with clindamycin. However, in the mutant system, binding of clindamycin stabilizes some nucleotides. Again, this is probably due to the different binding mode of clindamycin in the mutant.

Table S3 also classifies the nucleotide pairs according to the nucleotide edges that are involved in the interactions (following the Leontis and Westhof classification [43, 44], Fig. S5). Not only the WC-edges are involved but also Hoogsteen and sugar edges, which is expected since the binding pocket is not a regular duplex but has a varied architecture.

Figure S6 presents two types of interactions between G2505 and G2581 observed only in the WT trajectories (on average for 36 ± 6% of simulation time). These two nucleotides interact also with U2506. The presence of the clindamycin, as well as the A2058G mutation, blocks the formation of this interaction.

### Stacking interactions

Apart from hydrogen bonds, some stacked bases were also observed within clindamycin binding cavity. Figure S7 shows selected stacking patterns in the WT and MUT trajectories. Quantification of stacking energetics for the bases in the clindamycin binding pocket is shown in Table S4. According to the van der Waals energy criterion (see Methods), which corresponds to geometric criterion, the base 2058 stacks with A2059 regardless of the purine type and both in the free and complexed ribosome fragments.

Overall, the stacking pattern in the clindamycin cavity is more similar in all simulations in comparison to the hydrogen bond pattern which differs among simulation types. The main difference is that in the free MUT system, G2505 stacks with U2506 and this stacking interaction was not observed in the WT system.

## Conclusions

We performed MD simulations of clindamycin binding site in the bacterial ribosome, including the A2058G mutation in 23S RNA. There is experimental evidence that the A2058G mutation makes bacteria resistant to clindamycin [48, 49]. The simulations at atomic resolution and in explicit solvent were carried out for the ribosome fragment that included the clindamycin binding site.

We observed that clindamycin is more internally flexible in the WT binding site structure than in the A2058G mutated one (Fig. 5), which corroborates with the fact that clindamycin may acquire various conformers. Only in the WT ribosome fragment did we observe intramolecular conformational change in clindamycin around the C7-N1-C10-C11 dihedral angle (Fig. 6). Two clindamycin conformers differing in this dihedral were previously observed in the ribosome crystal structures in complexes with clindamycin [21, 22], in docking studies [25], and also by us in the simulations of clindamycin without the ribosome environment [19]. The lack of conformational change of clindamycin inside the mutant is probably due to its different binding mode and spatially smaller cavity in the mutated complex (Fig. 11).

The A2058G mutation changes the interactions of clindamycin with the 2058 nucleotide but also with the neighboring A2059. G2058 makes more hydrogen bonds with clindamycin. This makes clindamycin in the mutant slightly shifted and leads to a slightly different mode of binding, which exposes the light of ribosome tunnel, otherwise closed if clindamycin binds to a mutant (Fig. 8).

In summary, we suggest that bacterial resistance for the A2058G mutant could arise from: (i) a smaller percentage of clindamycin binding-enabled conformations in the mutant and (ii) if bound to the mutant clindamycin does not obstruct the entrance to the polypeptide exit tunnel. However, future studies using some enhanced sampling techniques are needed to obtain a more complete picture of the accessible conformations in this site.

## Electronic supplementary material

Below is the link to the electronic supplementary material.

(PDF 4.69 MB)
