# Supplementary material for: Molecular dynamics simulations suggest why the A2058G mutation in 23S RNA results in bacterial resistance against clindamycin
Source: J Mol Model. 2018 Jul 3;24(8):191. doi: 10.1007/s00894-018-3689-5 (PMC6028897; doi:10.1007/s00894-018-3689-5)
Supplement: Supplementary file 1 — (PDF 4.69 MB) [file 894_2018_3689_MOESM1_ESM.pdf]

Molecular dynamics simulations suggest why the A2058G mutation in 23S RNA results in bacterial resistance against clindamycin – Supporting Material

Katarzyna Kulczycka-Mierzejewska[1] Joanna Sadlej[2]
Joanna Trylska[3*]

[1] Interdisciplinary Centre for Mathematical and Computational Modelling, University of Warsaw, Warsaw, Poland

[2] Faculty of Chemistry, University of Warsaw, Warsaw, Poland

[3] Centre of New Technologies, University of Warsaw, Warsaw, Poland

[*] joanna@cent.uw.edu.pl

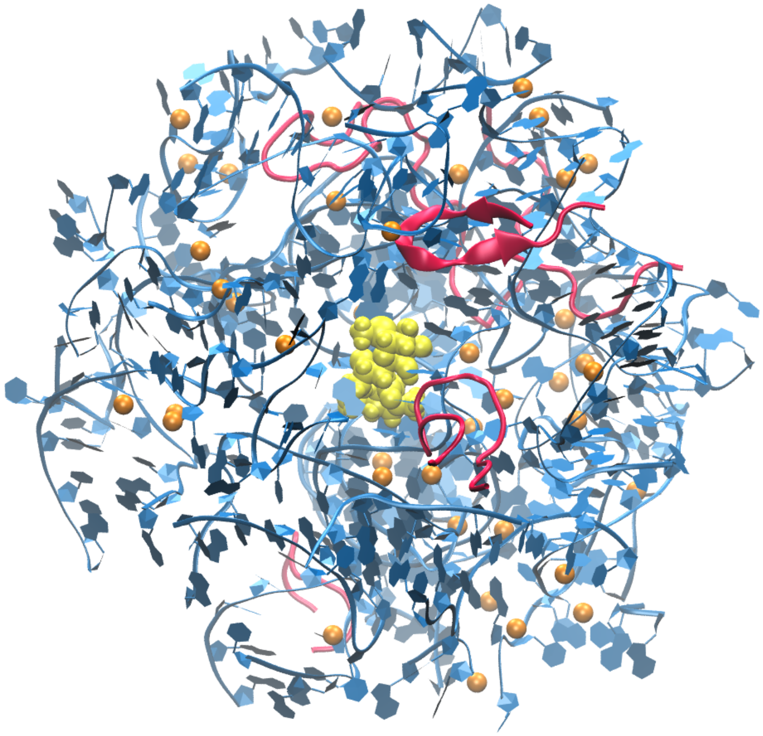

Fig. S1: The starting structure of the simulated fragment of the 50S ribosomal subunit: rRNA (blue ribbons), ribosomal proteins (red cartoon) and Mg^{2+} (orange spheres), clindamycin (yellow spheres). The sphere was cut 20 Å around clindamycin (the detailed description is given in the main text in Section *Methods*.

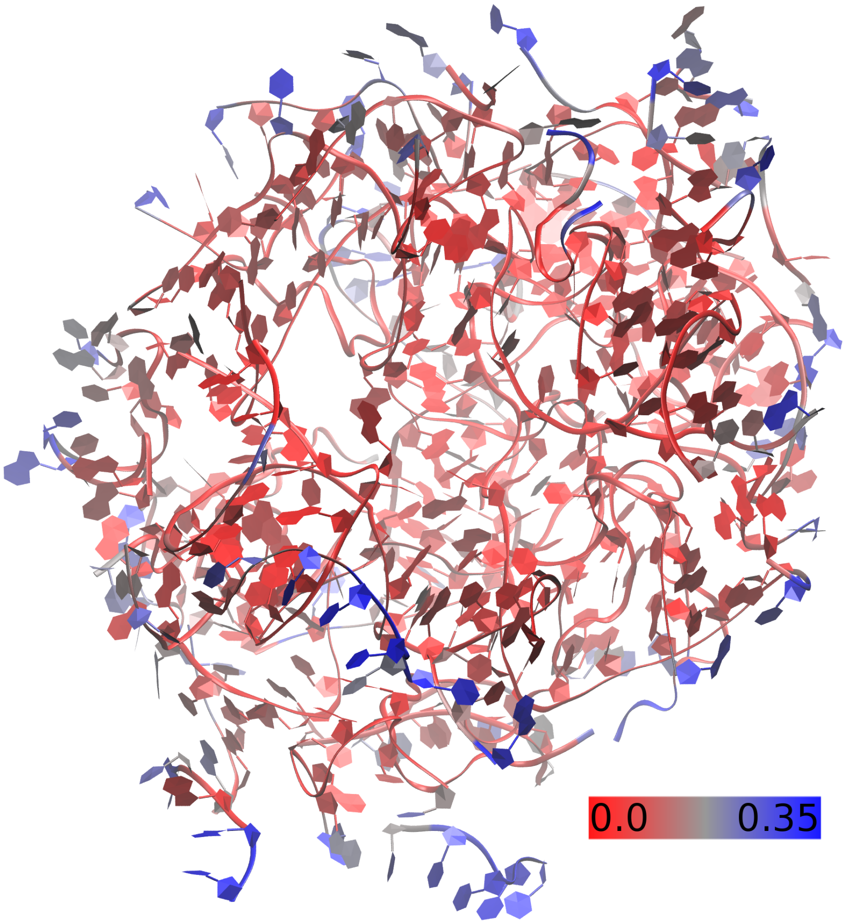

Fig. S2: The structure of the simulated fragment of the 50S ribosomal subunit colored according to restraints used in MD production simulations. The colours represent the force constant k values in kcal/(mol $\cdot$ Å^2) of the restraints; the color scale shown in the inset is in kcal/(mol $\cdot$ Å^2).

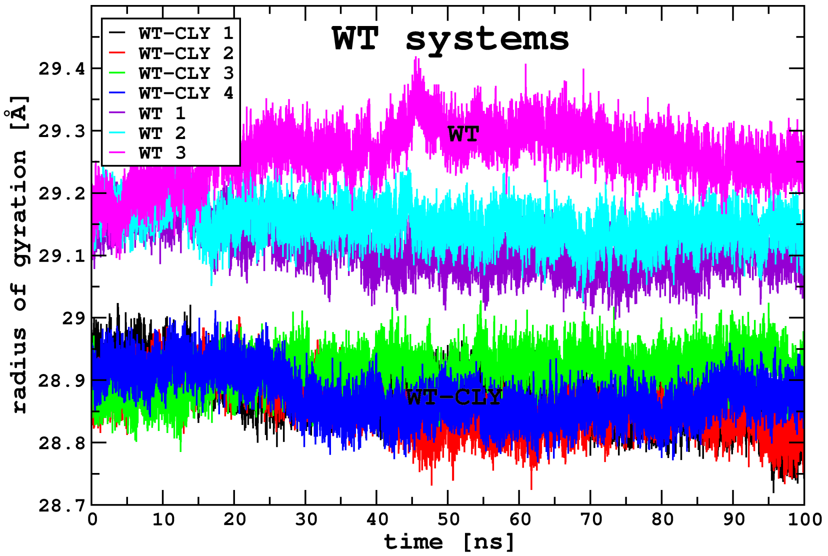

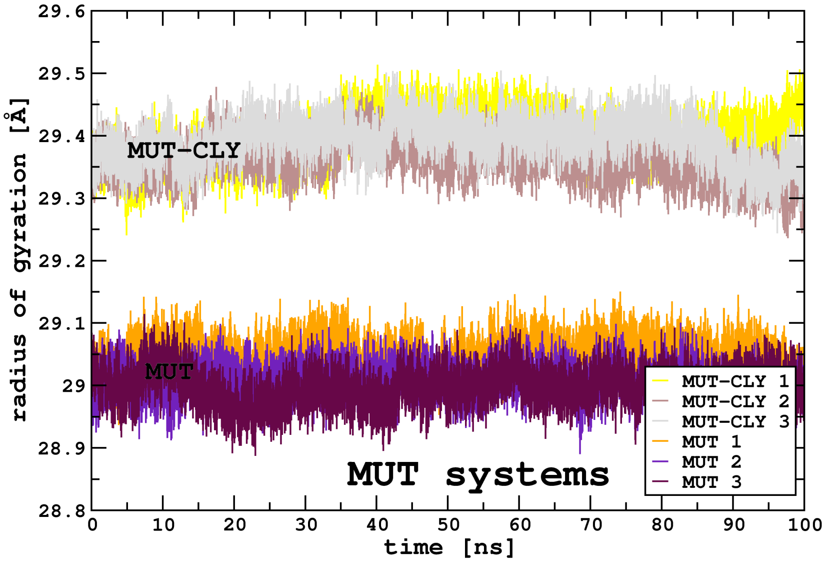

Fig. S3: The radius of gyration [Å] calculated for the C_α and P atoms with respect to the starting crystal structure plotted as a function of the production simulation time. The starting values of R_G were 29.15 Å for the WT, 29.00 Å for MUT, 28.91 Å for WT–CLY, and 29.35 Å for MUT–CLY system. For the description of the simulation types see Table 1 in the main text.

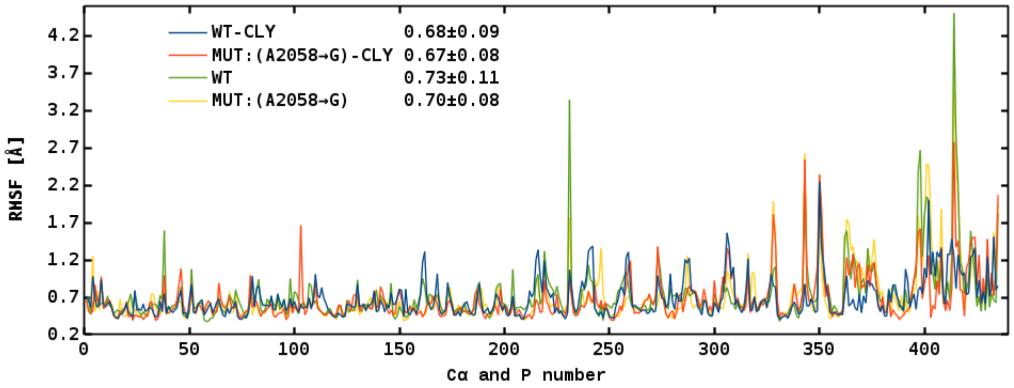

Fig. S4: RMSF [Å] calculated in the production simulations for the C_α and P atoms with averages (and standard deviations) shown in the legend. In all simulations the terminal and solvent exposed fragments are the most flexible, e. g., the peaks at ca. 400th position come from a short protein chain without any secondary structure and the green peak at ca. 230th position comes from a terminal nucleotide in our system (C2073 of *E. coli* 23S RNA numbering).

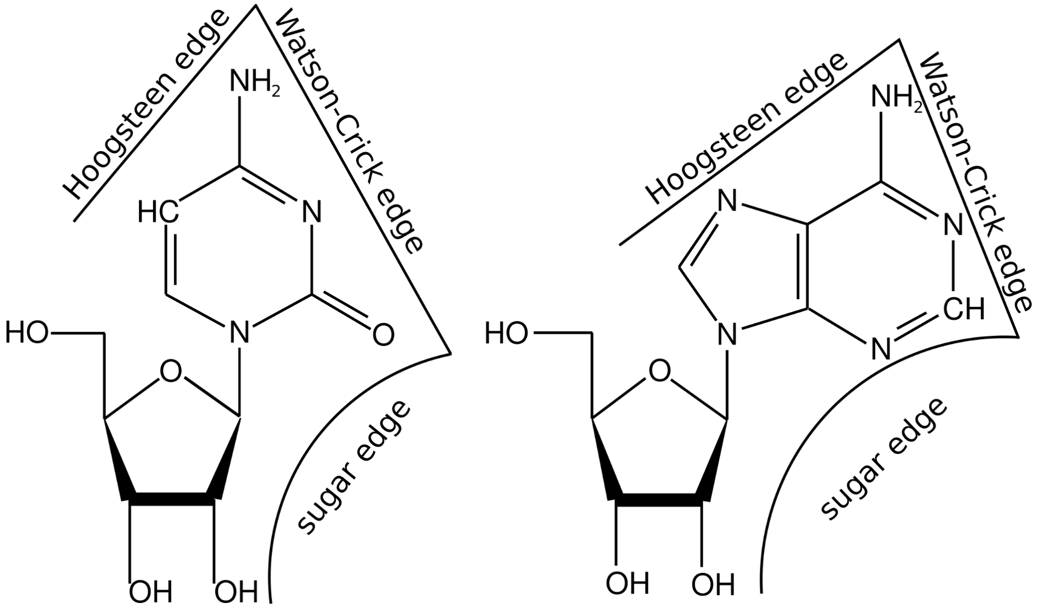

Fig. S5: The definition of the nucleotide edges according to Lescoute, A. and Westhof, E. Nucl. Acids Res. 34(22), 6587–6604 (2006), shown for cytidine and adenosine.

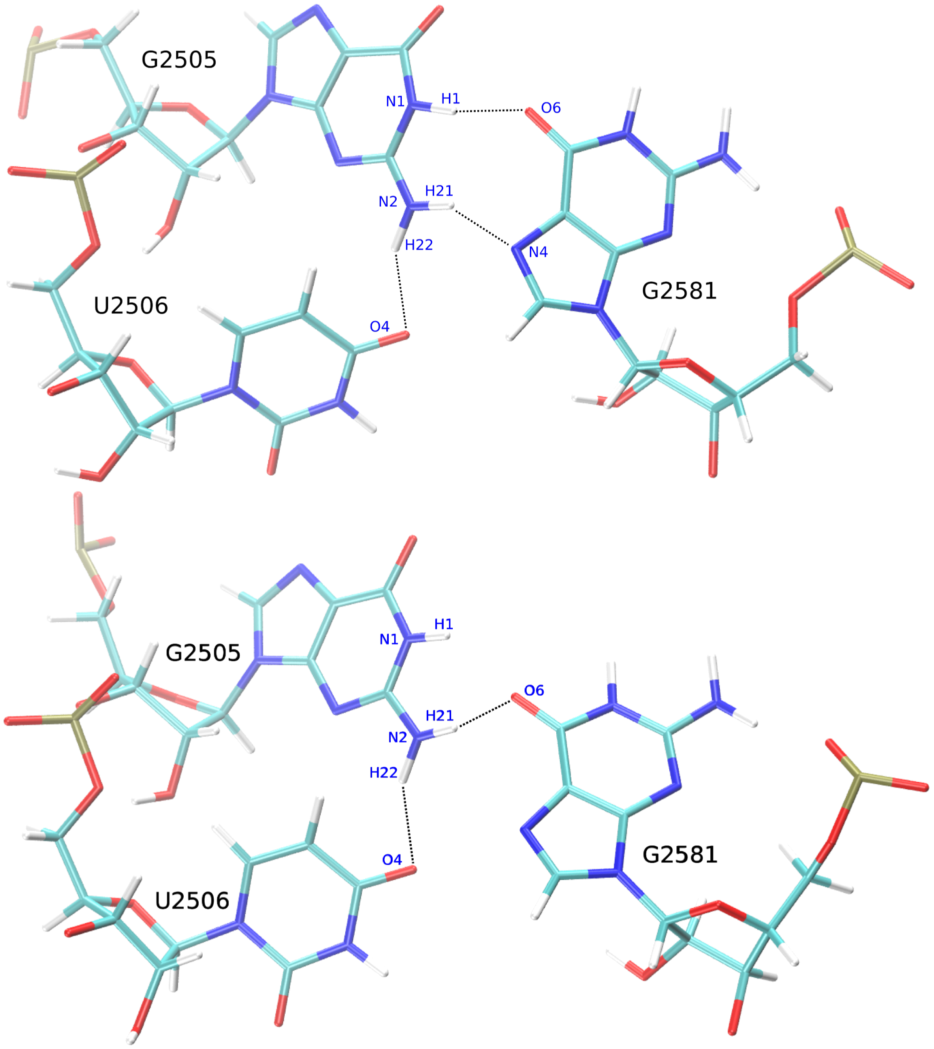

Fig. S6: Snapshots from the WT trajectory at the starting point (top) and at ca. 50 ns (bottom) showing the differences in hydrogen bond network of G2505 and G2581 nucleotides. During MD simulations the G2505···G2581 pair switches between interactions via the WC edges and via WC/Hoogsteen edge (see Figure **S5** and Table **S3**).

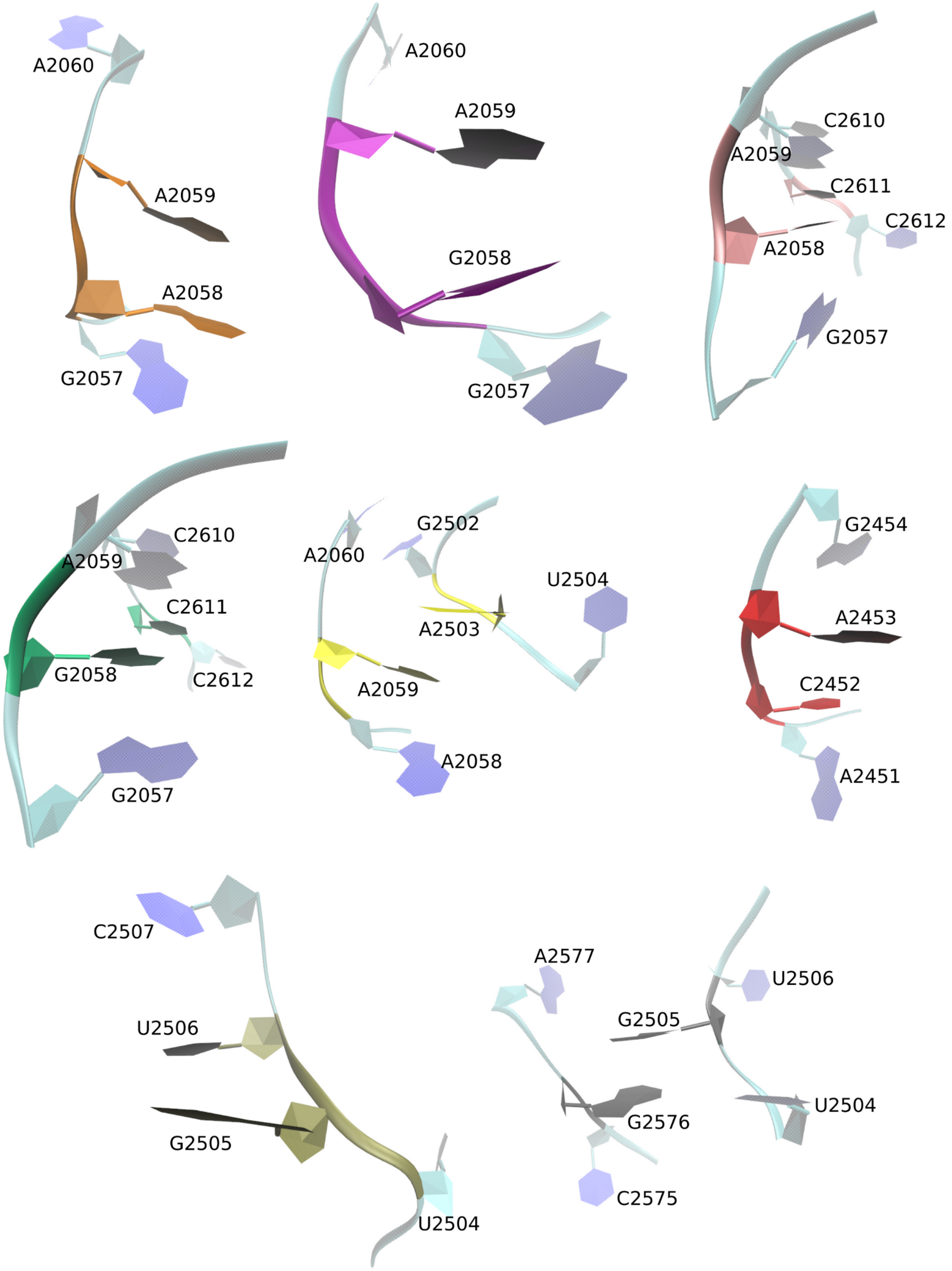

Fig. S7: Simulation snapshots from the free WT and MUT trajectories showing selected stacked nucleotides described in Table **S4**.

Tab. S1: The average RMSF values with standard deviations [Å] calculated for heavy atoms of selected nucleotides. The values were further averaged over all simulations of each type.

nucleotide	simulation types			
	WT-CLY	MUT-CLY	WT	MUT
A/G2058	1.21± 0.17	0.68± 0.14	0.97± 0.25	0.90± 0.20
A2059	1.31± 0.30	0.61± 0.08	0.86± 0.20	0.98± 0.26
C2452	0.89± 0.22	0.51± 0.09	0.57± 0.09	0.65± 0.07
A2503	1.31± 0.17	1.14± 0.43	0.71± 0.08	0.84± 0.11
G2505	1.16± 0.41	0.81± 0.26	1.26± 0.40	0.79± 0.09
U2506	1.24± 0.26	0.86± 0.17	0.87± 0.20	0.72± 0.06

Tab. S2: Average differences between the centers of massess (c.o.m.) of selected nuclebases and clindamycin between MUT–CLY and WT–CLY (2nd column) and between MUT and WT (3rd column) trajectories.

c.o.m. distance between:	c.o.m. difference between: [Å]	
	(MUT-CLY − WT-CLY)	(MUT − WT)
A2450–U2585	4.1 ± 0.8	-2.8 ± 0.9
A2451–U2585	3.9 ± 0.9	-4.4 ± 0.2
A2506–U2584	3.5 ± 0.8	-1.0 ± 0.5
U2451–CLY	2.0 ± 0.6	–
U2584–CLY	6.6 ± 0.8	–
U2585–CLY	4.3 ± 0.9	–

Tab. S3: The description of selected hydrogen bonds considering nucleotide edges through which the hydrogen bond was formed, together with the percent of simulation time the bonds were formed in the trajectory (averaged over all simulations of the given type, with the threshold for occurrence of 10%). The edges were assigned according to nomenclature shown in Figure **S5**. The * asterisk in the table indicates that it was impossible to assign the edge uniquely; this happens if only one hydrogen bond is formed between nucleobases and involves the indistinguishable hydrogen of participating groups.

nucleotide pairs	interacting edges	configuration	% of sim. time
	**WT–CLY**		
G2505-U2506	Sugar/Hoogsteen	*cis*	26%
U2506-U2584	WC/WC*Sugar	*cis*	29%
	**MUT–CLY**		
G2505-U2506	WC*Sugar/WC*Hoogsteen	*cis*	41%
G2505-C2611	WC*Hoogsteen/WC*Hoogsteen	*cis*	61%
U2506-C2507	Sugar/Hoogsteen	*cis*	44%
U2506-C2610	WC*Hoogsteen/WC*Hoogsteen	*cis/trans*	20%
	**WT**		
C2452-U2500	WC*Hoogsteen/WC*Hoogsteen	*cis*	25%
C2452-U2504	WC/WC	*cis*	93%
G2505-U2506	WC*Sugar/WC*Hoogsteen	*cis*	73%
G2505-G2581	WC/WC	*cis*	36%
G2505-G2581	WC/Hoogsteen	*cis*	25%
G2505-G2581	WC/WC*Hoogsteen	*cis*	21%
U2506-G2586	WC/WC	*cis*	77%
U2506-G2583	WC*Sugar/WC	*cis*	14%
U2506-C2610	WC*Hoogsteen/WC*Hoogsteen	*cis*	26%
	**MUT**		
C2452-U2500	WC*Hoogsteen/WC*Hoogsteen	*cis*	71%

Tab. S4: List of stacked nucleotides with the averaged vdW stacking energy (in kcal/mol) and percentage of simulation time the interaction was present. Selected snapshots of stacking nucleotides are shown in Fig. **S7**.

vdW energy	% of sim. time	simulation type
**A/G2058–A2059**		
-3.8±1.2	82	WT-CLY
-4.7±0.9	95	MUT-CLY
-5.1±1.0	99	WT
-3.0±0.9	94	MUT
**A/G2058–C2611**		
-2.5±0.9	81	WT-CLY
-3.4±0.7	96	MUT-CLY
-2.1±0.6	92	WT
-3.9±0.9	98	MUT
**A2059–A2503**		
-3.9±1.6	64	WT-CLY
-5.0±0.9	87	MUT-CLY
-6.0±1.0	62	WT
-4.9±0.8	88	MUT
**C2452–A2453**		
-3.5±1.2	98	WT-CLY
-4.3±1.0	100	MUT-CLY
-5.3±0.8	100	WT
-4.7±0.7	100	MUT
**G2505–U2506**		
-2.2±0.9	45	WT-CLY
-1.4±0.4	33	MUT-CLY
-	-	WT
-4.4±0.9	93	MUT
**G2505–G2576**		
-6.0±1.4	93	WT-CLY
-5.6±1.2	100	MUT-CLY
-7.7±0.7	100	WT
-	-	MUT
**U2506–C2507**		
-1.8±0.8	38	WT-CLY
-2.0±0.8	53	MUT-CLY
-3.4±0.5	96	WT
-2.2±0.7	56	MUT
